# To Refer or Not to Refer in Teledermoscopy: Retrospective Study

**DOI:** 10.2196/40888

**Published:** 2022-09-23

**Authors:** Esmée Tensen, Femke van Sinderen, Marcel W Bekkenk, Monique W Jaspers, Linda W Peute

**Affiliations:** 1 Department of Medical Informatics Amsterdam UMC location University of Amsterdam Amsterdam Netherlands; 2 Amsterdam Public Health, Digital Health Amsterdam Netherlands; 3 Ksyos Health Management Research Amsterdam Netherlands; 4 Department of Dermatology Amsterdam UMC location Vrije Universiteit Amsterdam Amsterdam Netherlands; 5 Cancer Center Amsterdam Amsterdam Netherlands

**Keywords:** teledermoscopy, dermoscopy, telemedicine, telehealth, triage, general practitioner, GP, general practice, family doctor, family physician, unnecessary referrals, refer, referral, skin, lesion, specialist, physician communication, diagnostic, interprofessional, diagnose, diagnosis, dermatology, dermatologist

## Abstract

**Background:**

Challenges remain for general practitioners (GPs) in diagnosing (pre)malignant and benign skin lesions. Teledermoscopy (TDsc) supports GPs in diagnosing these skin lesions guided by teledermatologists' (TDs) diagnosis and advice and prevents unnecessary referrals to dermatology care. However, the impact of the availability of TDsc on GPs’ self-reported referral decisions to dermatology care before and after the TDsc consultation is unknown.

**Objective:**

The objective of this study is to assess and compare the initial self-reported referral decisions of GPs before TDsc versus their final self-reported referral decisions after TDsc for skin lesions diagnosed by the TD as (pre)malignant or benign.

**Methods:**

TDsc consultations requested by GPs in daily practice between July 2015 and June 2020 with a TD assessment and diagnosis were extracted from a nationwide Dutch telemedicine database. Based on GP self-administered questions, the GPs’ referral decisions before and their final referral decision after TDsc consultation were assessed for (pre)malignant and benign TD diagnoses.

**Results:**

GP self-administered questions and TD diagnoses were evaluated for 6364 TDsc consultations (9.3% malignant, 8.8% premalignant, and 81.9% benign skin lesions). In half of the TDsc consultations, GPs adjusted their initial referral decision after TD advice and TD diagnosis. Initially, GPs did not have the intention to refer 67 (56.8%) of 118 patients with a malignant TD diagnosis and 26 (16.0%) of 162 patients with a premalignant TD diagnosis but then decided to refer these patients after the TDsc consultation. Furthermore, GPs adjusted their decision from referral to nonreferral for 2534 (74.9%) benign skin lesions (including 676 seborrheic keratosis and 131 vascular lesions).

**Conclusions:**

GPs adjusted their referral decision in 52% (n=3306) of the TDsc consultations after the TD assessment. The availability of TDsc is thus of added value and assists GPs in their (non)referral for patients with skin lesions to dermatology care. TDsc resulted in referrals of patients with (pre)malignant skin lesions that GPs would not have referred directly to the dermatologist. TDsc also led to a reduction of unnecessary referrals of patients with low complex benign skin lesions (eg, seborrheic keratosis and vascular lesions).

## Introduction

In the Netherlands, patients that are concerned about their skin lesion visit their general practitioner (GP) for advice. GPs assess the skin lesions and decide if a wait-and-see policy is justified, if they can manage the skin condition themselves in their practice, or if the patient should be referred to a dermatologist. In this way, GPs serve as gatekeepers and play a key role in deciding whether a patient is referred to Dutch dermatology care. However, GPs seem to find distinguishing between benign and malignant skin lesions a difficult task [1,2]. As a result, GPs frequently refer patients with suspicious skin lesions to a dermatologist that turn out to be benign (eg, seborrheic keratosis, vascular lesions, and benign nevus) [1-4]. These mild benign skin conditions can be managed by the GP in the primary care setting, and no clinical or surgical dermatological intervention is required [1,2]. Teledermoscopy (TDsc) can provide diagnostic support to GPs to accurately triage people with suspicious skin lesions [5-8]. With TDsc, more urgent cases can be correctly referred to a dermatologist, while unnecessary referrals of people with nonsuspicious skin lesions who can be managed in primary care are avoided [5-11].

In general, previous TDsc evaluation studies in primary care settings included all eligible patients with suspicious (pigmented) skin lesions, patients who GPs regularly intend to refer, or patients who were already referred to a hospital or lesion clinic [5-7,9-11]. In addition, these previous TDsc studies were often carried out in a study setting where the feasibility of TDsc was examined with a simulated TDsc service that was not yet integrated into GP daily practice. Furthermore, in some of these TDsc studies, the GP did not act as a gatekeeper, the referral decision was made by a (tele)dermatologist and not by a GP, or the photos of the skin lesions were not acquired by the GP themself but, for example, by a trained nurse (also called a melanographer) [6-11].

In the Netherlands, TDsc has been integrated into GP practices nationwide since 2009 by a Dutch telemedicine provider (Ksyos) and is fully reimbursed by Dutch health insurance companies [12]. The Ksyos TDsc service is unique compared to other worldwide TDsc services in primary care because this service (1) is implemented in GP general practice, (2) asks GPs to enter their initial referral decision in the Ksyos system at the start of a TDsc consultation request, and (3) asks GPs to enter their final referral decision in the system after receiving the digital assessment of the teledermatologist (TD) based on the overview, detailed, and dermoscopic images. Our previously performed TDsc evaluation in Dutch GP practices in the same context and the same Dutch TDsc system showed that the GPs adjusted their referral decision after the TD assessment in 3722 (53.3%) of the 6977 TDsc consultations [13].

Previous TDsc studies in other settings investigated common TD-provided telediagnoses and the percentage of patients for whom, due to TDsc, a physical referral to a dermatologist could be avoided [5-11]. However, these studies did not focus on patients who would initially not have been referred by the GP without the availability of TDsc. Nor did they aim to assess whether the GP’s initial decision to refer or not refer a patient before the TDsc consultation changed after the TD assessment for skin lesions diagnosed by the TD as malignant, premalignant, or benign.

Therefore, for these diagnosis groups, the impact of the availability of TDsc on the GPs’ referral decisions to dermatology care is still unknown. Therefore, this study assessed and compared GPs’ self-reported initial referral decisions before TDsc with their final referral decisions after TDsc for (pre)malignant and benign TD-diagnosed skin lesions.

## Methods

### Setting and TDsc Process Description

In the Ksyos-secured TDsc digital health record system, a GP starts the TDsc process with a standardized consultation request and uploads the obtained (detailed, overview, dermoscopic) images of a patient’s skin lesion. After a GP has filled in patient information, such as year of birth, sex, prehistory of skin cancer, structured anamnesis, optional provisional diagnosis, and additional notes, the GP sends the TDsc request to a TD for review. The TD then provides a primary diagnosis (a mandatory and an optional differential diagnosis) in a text entry field and referral recommendations, which may include advice for the GP on the patient management plan.

Further, a GP is asked to answer 2 similar nonmandatory self-administered questions: (1) “Would you have referred this patient if TDsc was not available?” and (2) “Are you still referring this patient to the dermatologist?”. These questions, which are embedded in the Ksyos system by default, retrieve information about (1) the GP’s initial decision to refer a patient to a dermatologist (Yes, No) when sending the TDsc consultation request to a TD and (2) the GP’s final referral decision (Yes, No) at the time of closing the TDsc consultation after the TD assessment.

As of July 2015, the Ksyos system generates an ICD-10 (International Classification of Diseases, 10th revision) [14] code by which diagnoses provided by TDs in TDsc consultations are automatically classified. Instead of describing the primary diagnosis in a free text entry field, TDs can also choose 1 of 3 icon buttons; no diagnosis (ICD-10 code: R69), no abnormalities (ICD-10 code: R68.8), or nonassessable (−).

### Ethical Considerations

No ethical approval was required to evaluate the number of TDsc consultations, since all GPs gave permission through a contract with Ksyos to monitor TDsc quality with these self-administered questions.

### Study Design

For this retrospective database study, TDsc consultations requested by GPs between July 2015 and June 2020 were included in the data analysis. Next, consultations with missing values were excluded. Missing values in the database were defined as a TD report of “no diagnosis” (R69), “no abnormalities” (R68.8), or “nonassessable” (−), or if a GP had not answered both self-administered questions. Data acquired included (1) answers to the GP self-administered questions on referral of a patient to a dermatologist and (2) diagnosis provided by TD during the TDsc consultation. Optional differential diagnoses provided by the TD were omitted from this study. Types of cameras or digital dermoscope used to obtain the images were unknown.

The GP self-administered questions were used to define whether the GPs had or had not adjusted their initial decision to refer a patient to a dermatologist after reviewing the advice and diagnosis of the TD.

In this study, 3 diagnosis groups were defined based on the TD diagnoses and the corresponding ICD-10 codes: malignant, premalignant, and benign. The histopathology and face-to-face diagnoses were not available in our study. Malignant skin lesions included all malignant neoplasms (ICD-10 codes C00-C97) such as melanoma, basal cell carcinoma, and squamous cell carcinoma. Premalignant skin lesions were defined as a separate group and included in situ neoplasms (ICD-10 codes D00-D09), other specified epidermal thickening (ICD-10 code L85.8; eg, keratoacanthomas), and actinic keratosis (ICD-10 code L57.0). Benign skin lesions included the remaining ICD-10 diagnoses. In this group, we specifically focused on seborrheic keratosis (ICD-10 code L82) and vascular lesions (ICD-10 codes D18, I78.1). For each diagnosis group, the GP self-reported initial and final referral decisions were analyzed.

## Results

### Overall Cohort

**Figure 1:**
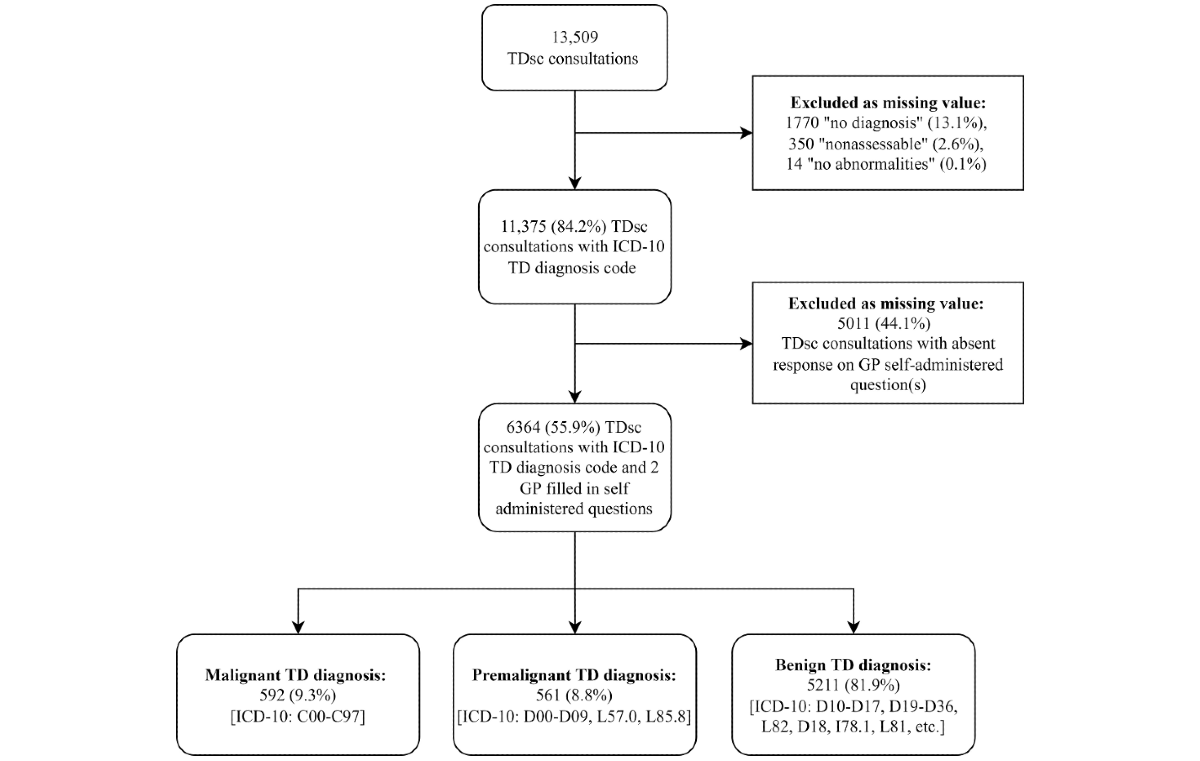
Flowchart of teledermoscopy (TDsc) consultations requested by general practitioners (GPs) between July 2015 and June 2020 as included in our study. ICD-10: International Classification of Diseases, 10th revision; TD: teledermatologist.

In total, 13,509 TDsc consultations requested by 1185 GPs between July 2015 and June 2020 were provided with a diagnosis by 140 TDs. Of these, 1770 (13.1%) were assessed by the TD as “no diagnosis,” 14 (0.1%) as “no abnormalities,” and 350 (2.6%) as “nonassessable.” Moreover, 5011 (44.1%) TDsc consultations had an absent response on the GP self-administered question(s) and were therefore excluded as a missing value from the data set (Figure 1). For 6364 (55.9%) of the 11,375 TDsc consultations with an ICD-10 TD diagnosis code, both nonmandatory self-administered questions were answered by the GP. According to the TD diagnosis, this consisted of 592 (9.3%) skin lesions in the malignant diagnosis group, 561 (8.8%) in the premalignant diagnosis group, and 5211 (81.9%) in the benign diagnosis group. Overall, benign skin lesions were the most frequently reported diagnosis by the TDs.

Among the group of malignant diagnoses, the most common were basal and squamous cell carcinoma (n=415, 70.1%) followed by malignant melanoma (n=172, 29.1%). The most commonly provided diagnosis in the premalignant diagnosis group was actinic keratosis (ICD-10 code L57.0; n=434, 77.4%). Among the group of benign diagnoses, the most common was melanocytic nevus (ICD-10 code D22; n=2571, 49.3%), followed by seborrheic keratosis (n=1221, 23.4%).

### GPs’ Referral Decision Based on Self-Administered Questions

In 3306 (51.9%) TDsc consultations, the GPs adjusted their referral decision (Yes-No, No-Yes) after the TD assessment (Table 1). For the malignant diagnosis group, GPs indicated that they would initially not have referred 118 (19.9%) patients without TDsc. For 67 (56.8%) of these 118 patients with a malignant TD diagnosis, the GPs adjusted their initial referral decision and referred the patient after TDsc consultation.

In the premalignant diagnosis group, the GPs indicated that they would not have referred for 162 (28.9%) patients without TDsc. For 26 (16.0%) of these 162 patients with a premalignant TD diagnosis, the GPs changed their decision from nonreferral to referral.

In the benign diagnosis group, 3384 (64.9%) patients with benign skin lesions, of which 784 (64.2%) had seborrheic keratosis and 163 (70.6%) had vascular lesions, would have been referred by the GP without the availability of TDsc. The TD-provided benign diagnoses resulted in a change of the GPs’ decision from referral to nonreferral for 2534 (74.9%) patients. More specifically, GPs adjusted their referral decision to nonreferral after the TD assessment for 676 (86.2%) patients with a seborrheic keratosis TD diagnosis and 131 (80.4%) patients with a vascular lesion TD diagnosis. In addition, the group of “other benign skin lesions” included benign nevi as well as ICD-10 codes for eczema and insect bites.

**Table 1:** Number of teledermatologists (TD) diagnoses for the general practitioner (GP) self-administered questions.

Self-administered questions		Malignant skin lesions (N=592), n (%)	Premalignant skin lesions (N=561), n (%)	Benign skin lesions, n (%)				Total TDsc^a^ consultations (N=6364), n (%)
				Seborrheic keratosis (N=1221)	Vascular lesions (N=231)	Other benign skin lesions (N=3759)	Total benign skin lesions (N=5211)	
**Q1^b^=Yes**		474 (80.1)	399 (71.1)	784 (64.2)	163 (70.6)	2437 (64.8)	3384 (64.9)	4257 (66.9)
	Q2^c^=Yes	353 (74.5)	122 (30.6)	108 (13.8)	32 (19.6)	710 (29.1)	850 (25.1)	1325 (31.1)
	Q2=No	121 (25.5)	277 (69.4)	676 (86.2)	131 (80.4)	1727 (70.9)	2534 (74.9)	2932 (68.9)
**Q1=No**		118 (19.9)	162 (28.9)	437 (35.8)	68 (29.4)	1322 (35.2)	1827 (35.1)	2107 (33.1)
	Q2=Yes	67 (56.8)	26 (16.0)	36 (8.2)	9 (13.2)	236 (17.9)	281 (15.4)	374 (17.8)
	Q2=No	51 (43.2)	136 (84.0)	401 (91.8)	59 (86.8)	1086 (82.1)	1546 (84.6)	1733 (82.2)

^a^TDsc: teledermoscopy.

^b^First GP self-administered question: Would you have referred this patient if TDsc was not available?

^c^Second GP self-administered question: Are you still referring this patient to the dermatologist?

## Discussion

### Principal Results

This retrospective study assessed the impact of the availability of TDsc on GPs’ self-reported decisions to refer patients to the dermatologist. GPs’ self-reported initial referral decisions before the TDsc consultation were compared with their referral decisions after the TDsc consultation for skin lesions diagnosed by the TD as (pre)malignant or benign. This study showed that for these lesions, GPs adjusted their initial referral decision after the TD assessment in half of the TDsc consultations.

For 26 (16%) of 162 patients with a premalignant TD diagnosis and for 67 (56.8%) of 118 patients with a malignant TD diagnosis, GPs adjusted their referral decision after the TDsc consultation from nonreferral to referral. Therefore, without the availability of TDsc, GPs would not have referred these patients with (pre)malignant TD diagnoses directly to the dermatologist.

Furthermore, if the TD provided the diagnosis seborrheic keratosis, GPs adjusted their referral decision in 676 (86.2%) of the 784 TDsc consultations from referral to nonreferral. For the TD diagnosis of vascular skin lesions, GPs adjusted their referral decision in 131 (80.4%) of the 163 TDsc consultations from referral to nonreferral. Therefore, without the availability of TDsc, GPs would have referred these patients with benign skin lesions to a dermatologist.

### Comparison With Prior Work

In a Belgian TDsc study, which included all patients with suspicious skin lesions for TDsc, regardless of whether the GPs intended to refer the patients, GPs photographed all skin lesions as part of the TDsc consultation [10]. The vast majority of these skin lesions were assessed by the TD as benign (n=91, 86.7%), malignant (n=8, 7.6%), and uncertain classified diagnoses (n=6, 5.7%). These percentages are comparable with the TD-assessed skin lesions in our TDsc study, in which 81.9% (n=5211) were benign, 8.8% (n=561) were premalignant, and 9.3% (n=592) were malignant.

In contrast to our study, a Danish and a Swedish TDsc study included only patients with suspicious skin lesions that the GPs, without the availability of TDsc, would have referred to the dermatologist [5,6]. All these patients were seen in-person by a dermatologist after the TDsc consultation. These studies reported that 27.7% (n=166) and 28.1% (n=229) of the skin lesions were diagnosed by the TD as (pre)malignant and 72.3% (n=434) and 71.9% (n=587) were diagnosed as benign, respectively. For the same group of patients in our study, where the GPs indicated that they initially would have referred the patient to the dermatologist, we found a slightly lower percentage of patients with (pre)malignant diagnosed skin lesions (n=873, 20.5%) and a slightly higher percentage of patients with benign diagnosed skin lesions (n=3384, 79.5%).

In these 3 TDsc studies, all patients with suspicious skin lesions, along with patients that the GPs initially would have referred for a physical dermatological consultation, were included. By contrast, in our study, which was performed in daily general practice, GPs acted as gatekeepers to dermatology care. GPs decided themselves whether to apply TDsc, justify a wait-and-see policy, manage the skin condition themselves, or refer the patient to a dermatologist.

Previous findings show that TDsc is especially valuable for the triage of patients with benign skin lesions. The relatively fast TD assessment of skin lesions diagnosed as evidently benign reassures and avoids nervous waiting for both patients and practitioners [15,16]. TDsc also releases the burden on dermatology care since most patients with benign skin lesions can be managed appropriately in GP practice without the need for a physical referral to a dermatologist [5-11]. Moreover, this means that dermatologists can allocate more time to the treatment of patients with complex skin lesions. In addition, patients with severe (pre)malignant skin lesions who need an urgent in-person dermatological evaluation will have improved access to the dermatologist due to the availability of TDsc [5,10,11].

Remarkably, the GPs in our study also applied TDsc to request TD advice concerning nonpigmented benign diagnoses, such as eczema, psoriasis, and insect bites, which is in accordance with 2 other TDsc studies in a virtual lesion clinic and primary health care center setting [9,10]. This implies that GPs also use TDsc as a diagnostic tool to request advice from the TD regarding the management of nonpigmented skin lesions. Dermatologists do not need a dermoscopic photo to assess these types of skin lesions. However, we could not check whether the GPs uploaded a dermoscopic photo for these nonpigmented skin lesions in the TDsc consultation.

The TDsc service evaluated in our study is unique compared to other systems because it asks GPs to enter their initial referral decision at the start of the TDsc consultation request and their final referral decision after the TDsc consultation. In a retrospective TDsc study by our research group 5 years ago in the same nationwide context and with the same Dutch TDsc system, we found that the GPs adjusted their initial referral decision after TDsc in half of the consultations [13]. GPs thus still frequently change their referral decision after a TDsc consultation, which could be because they face difficulties when diagnosing skin lesions or discriminating between benign and malignant skin lesions [1,2,6,17]. GPs might lack this knowledge because dermatology education and skills such as biopsies are underrepresented in the Dutch medical and GP training curriculum [18]. GP residents must obtain this dermatological knowledge from their GP educators during the medicine internships, and this knowledge transfer might be limited. Furthermore, in the Netherlands, most patients from GP primary care are referred to dermatology secondary care [19]. This again addresses the importance of TDsc as a tool to support GPs in primary practice in recognizing and gaining knowledge on skin lesions and by receiving instructions on patient management. Due to data migration and limitations in the Ksyos database, we could not check if both of our TDsc studies concerned the same GP population. Over the years, some GPs might have learned from the TD advice and applied TDsc less often. It is also possible that GPs who recently started applying the TDsc service frequently change their referral decisions. In any case, the frequently changing referral decisions of GPs emphasize the surplus value and need of TDsc to support GPs in their referral decisions of patients with skin lesions.

In an Italian study, GPs were also asked to assess photographed skin lesions and decide whether they would refer the patient to a dermatologist [3]. The authors of that study did not specify who took the photographs. After a 4-hour training on the classification and management of skin lesions, GPs were again asked about their referral decision for the same set of clinical images of skin lesions. GPs had to base their referral decision solely on the set of submitted clinical images without physically seeing the patients and skin lesions in their GP practice. Furthermore, the GPs did not receive a diagnosis or advice from the TD on which they could base their referral decision. In this Italian study, the number of nonmelanocytic benign skin lesions of patients whom GPs intended to refer to a dermatologist decreased significantly after training on the classification and management of skin lesions. This type of training could consist of e-learning, refreshers, and courses in the GP education programs regarding both taking dermoscopic images and recognizing pigmented skin lesions. Therefore, continuous training of GPs in the Dutch TDsc setting could potentially help reduce the number of referrals of patients with benign skin lesions [1].

### Strengths and Limitations

The strengths of this large retrospective study include that TDsc consultations were conducted in daily GP practice and were not simulated in a study setting. The GP referral decisions were noted both before and after the TDsc consultation, which allowed us to verify whether GPs adjusted their initial referral decisions after the TDsc consultation. In doing so, we gained insight into GP referral decisions for different diagnosis groups after the TD assessment in daily GP practice.

On the other hand, the first limitation of our study is that the TDs did not always report their diagnosis in the TDsc system and that we omitted data on the differential diagnosis. This might have resulted in an underestimation of the absolute number of (pre)malignant and benign diagnoses for which TDsc was applied by the GPs. It is possible that TDs were unable to provide a diagnosis because the GPs provided insufficient patient information in the TDsc consultation [20]. Furthermore, overview or dermoscopic photos taken by the GP may have been lacking in the TDsc consultation or may have been of insufficient quality [6,10]. The Ksyos TDsc system does not validate whether a dermoscopic photo of the skin lesion is available at all and if it is, whether the photo quality is sufficient. GPs can only retake the photos if they receive direct feedback from the TD and if the patient is present at the GP practice. In the future, an algorithm could be created into the TDsc system that assesses the photo quality and provides real-time, direct feedback to the GP if improvements are necessary. Showing instructions in the Ksyos TDsc system (eg, image quality checklist, guidelines on taking dermoscopic photos) could support GPs in filling in the TDsc consultation completely and ensure photos of sufficient quality and correct type (overview, detailed, dermoscopic) [21,22].

The second limitation of our study is that the GPs were not obliged to fill in the self-administered questions regarding their referral decisions; thus, these self-administered questions were not always filled in. For these TDsc consultations, we could not compare the GP referral decision before and after the TDsc consultation. In addition, we do not know if the GP interpreted these questions regarding their referral decision as originally intended in the TDsc system. The reasons why GPs decided not to physically refer patients with a TD-diagnosed (pre)malignant skin lesion are still unknown. Additionally, clinical follow-up data on these patients are lacking. The Dutch guideline for suspicious skin abnormalities recommends that GPs refer malignant skin lesions to the dermatologist [23]. We know from dermatology experience that it is possible for GPs to deviate from this guideline after contact with a dermatologist; for example, for elderly patients, if the GP is experienced in excision of lesions, if the excision has already been performed, or for superficial lesions that do not require invasive treatment. For premalignant diagnoses, TDs also have an important advisory role for GPs on how to treat patients. Referral of premalignant lesions is dependent on the condition (location, evolvement, etc). Consultations in which GPs initially did not plan to refer a benign lesion (after confirmation by TDsc) but then changed their decision could be due to an insistent patient. However, we know from dermatology experience that dermatologists have specialized treatment equipment available, such as laser and light therapy. It is also likely that GPs are not aware of these (aesthetic) treatment options before sending the TDsc consultation. The advantage of TDsc is that GPs are informed about these treatment options due to the TD response and that patients can receive this treatment.

The third limitation is that only the TDsc consultation data extracted from the Ksyos system were accessible for our study. Although Ksyos is the largest store-and-forward telemedicine provider in the Netherlands, the overall number of TDsc consultations in the Netherlands might be higher.

The fourth limitation is that no data concerning the histopathological diagnoses were available for our study. In practice, it is considered unethical to acquire, purely for research purposes, the histopathology of patients with benign skin lesions who not have been referred by the GP to the dermatologist (Q1=No and Q2=No; Q1=Yes and Q2=No). Vestergaard et al [6] showed in a pilot study that patients are reluctant to travel to the dermatologist for assessment of a supposedly benign skin lesion, and GPs are not willing to refer these patients to a dermatologist. Due to the retrospective nature of our study, it was not possible to obtain histopathological data of patients with skin lesions that were referred to dermatology care after the TDsc consultation (Q1=Yes and Q2=Yes; Q1=No and Q2=Yes). We can only presume that GPs would have immediately referred patients to the dermatologist if patients had skin lesions that were highly suspect of melanoma or dubious.

### Conclusions

This study showed that GPs adjusted their initial referral decision of patients with skin lesions in half of the studied TDsc consultations after the TD assessment. The availability of TDsc remains thus of added value to support GPs in gatekeeper health care systems in their decision to refer patients to a dermatologist for an in-person consultation. This study has shown that GPs initially did not intend to refer patients with (pre)malignant skin lesions for an in-person dermatological consultation and that the availability of TDsc aids in the referral of these patients. In addition, TDsc supports GPs in the prevention of unnecessary physical referrals to the dermatologist for patients with low complex benign skin lesions (eg, seborrheic keratosis and vascular skin lesions), easing the burden on dermatology care.

## Abbreviations

GP: general practitioner
ICD-10: International Classification of Diseases, 10th revision
TD: teledermatologist
TDsc: teledermoscopy
